# Comparative Analysis of Microsatellite and SNP Markers for Genetic Management of Red Deer

**DOI:** 10.3390/ani13213374

**Published:** 2023-10-31

**Authors:** Javier Pérez-González, Juan Carranza, Gabriel Anaya, Camilla Broggini, Giovanni Vedel, Eva de la Peña, Alberto Membrillo

**Affiliations:** 1 Biology and Ethology Unit, Veterinary Faculty, University of Extremadura, 10003 Caceres, Spain; 2 Wildlife Research Unit (UIRCP), University of Córdoba, 14071 Cordoba, Spain; jcarranza@uco.es (J.C.); b22ancag@uco.es (G.A.); z62brbrc@uco.es (C.B.); giove985@gmail.com (G.V.); evadelapenha@gmail.com (E.d.l.P.); b72depoa@uco.es (A.M.); 3 Department of Genetics, University of Cordoba, 14071 Cordoba, Spain; 4 Institute for Game and Wildlife Research (IREC), 13005 Ciudad Real, Spain; 5 Department of Specific Didactics, Faculty of Education Sciences, University of Cordoba, 14071 Cordoba, Spain

**Keywords:** *Cervus elaphus*, genetic diversity, genetic structure, inbreeding, multilocus heterozygosity

## Abstract

**Simple Summary:**

Population genetic structure and individual multilocus heterozygosity are vital for wildlife management. Traditionally, microsatellite markers have been used to estimate population genetic parameters, but single-nucleotide polymorphisms (SNPs) have gained popularity due to their greater measurement precision. This study compared genetic estimates at the population and individual levels using microsatellite and SNP markers in red deer (*Cervus elaphus*). The findings revealed correlations between parameters estimated with both markers that were associated with the level of genetic diversity and genetic differentiation. However, microsatellites showed lower accuracy in representing the distribution of genetic diversity among individuals.

**Abstract:**

The analysis of population genetic structure and individual multilocus heterozygosity are crucial for wildlife management and conservation. Microsatellite markers have traditionally been used to assess these genetic parameters. However, single-nucleotide polymorphisms (SNPs) are becoming increasingly popular. Our goal here was to determine to what extent SNPs can provide better insights than microsatellites into the overall genetic status and population genetic processes in the species. To this end, we genotyped 210 red deer (*Cervus elaphus*) in the Spanish wild population with both 11 microsatellites and 31,712 SNPs. We compared parameters related to population genetic structure and individual multilocus heterozygosity obtained with both types of markers. Our results showed correlations between parameters measured using both microsatellites and SNPs, particularly those related to the level of genetic diversity and genetic differentiation. However, we found notably lower precision of microsatellites in measuring the distribution of genetic diversity among individuals. We conclude that microsatellites can be used to monitor the overall genetic status and detect broad patterns in red deer populations. Nevertheless, the greater precision of SNPs in inferring genetic structure and multilocus heterozygosity leads us to encourage scientists and wildlife managers to prioritize their use whenever possible.

## 1. Introduction

Genetic structure plays a crucial role in wildlife management as it provides essential knowledge. The assessment of the spatial distribution of genetic diversity enables the identification of migration barriers [1], the connection between distant populations [2], eventual reductions of local variability [3], risks for the spread of infectious diseases [4,5], and aids in making inferences about population dynamics [6]. Therefore, genetic structure can be important in decision-making for population management, particularly in endangered species and faunal resources such as fisheries or game species [7,8,9,10,11,12,13]. Furthermore, genetic diversity can be associated with inbreeding and population viability [14], and heterozygosity-fitness correlations (HFCs) have been detected in endangered species and game populations [15,16]. Therefore, the assessment of the significance of local genetic diversity within the genetic structure of populations can help in the identification of inbreeding or in evaluating the feasibility of eventual restocking in wildlife management contexts.

Microsatellite markers (short tandem repeat loci or STRs) have been widely used in conservation genetics to estimate genetic structure [17,18] and detect HFCs [19]. These highly polymorphic loci not only allow for the detection of fine-scale genetic variation [20] but also serve as cost-effective and simple-to-use markers, rendering them accessible to researchers. Nevertheless, these markers have limitations, such as homoplasy [21], and they are prone to genotyping errors [22]. Moreover, studies on genetic structure and HFC using STR are often conducted with a relatively small number of markers [23]. Despite the high number of alleles per locus in microsatellites, the low number of loci can reduce the power of analyses related to the genetic structure of populations, such as analyses of spatial genetic patterns [24]. Moreover, the relationships between individual genetic diversity estimated from microsatellites and actual inbreeding are generally weak [25], and it has been suggested that the observed heterozygosity-fitness correlations (HFCs) may be due to linkage disequilibrium with fitness-linked loci rather than the effect of multilocus heterozygosity [26].

In recent years, the use of single-nucleotide polymorphisms SNPs to study genetic structure has increased [27,28,29]. Despite the fact that SNPs are mostly di-allelic, the advent of next-generation sequencing techniques has automated genome-wide SNP detection and favors the simplified genotyping of thousands of markers [30,31,32]. Consequently, a large number of SNPs significantly overcomes the higher number of alleles per locus in microsatellites and can greatly increase the power of analyses of genetic structure [24]. Moreover, analyses of genetic structure using candidate adaptive SNPs can offer insights into potential adaptive divergence among populations and the likelihood of local adaptations [33]. Furthermore, Fernández et al. [34] suggested that 2–3 SNPs per STR can suffice to achieve comparable power values between both types of markers in the genetic identification of individuals. Multilocus heterozygosity at thousands of SNP loci has been shown to be highly correlated with the inbreeding coefficient of known pedigrees [35]. Therefore, HFCs due to inbreeding depression are expected to be more easily detected by estimating genome-wide heterozygosity using SNPs than by using microsatellites.

The red deer (*Cervus elaphus*) is a widely distributed large mammal, ecosystem engineer, and an important game species in Europe [36]. Despite the abundance of red deer in most of its range, concerns have been raised regarding the preservation of their genetic composition in specific areas [37,38]. Furthermore, game management practices have the potential to impact the genetic structure of red deer populations through activities such as translocating individuals [39], preventing dispersal [40], modifying female aggregation patterns and polygyny degree [41], and altering population structures [42,43]. Additionally, low levels of genetic diversity might increase individuals’ susceptibility to pathogens [44] and might also have implications for antler development in stags [45].

Genetic structure analyses have been conducted in red deer populations across various European regions including the Iberian Peninsula [46,47,48,49], France [50,51], Luxemburg [52], Belgium [53], Scotland [20,54], Ireland [55], Germany [56,57], Denmark [58], Poland [59], Sweden [60], Norway [61], Czech Republic [62], Hungary [63], Croatia [64], Italy [65], or Greece [66]. Furthermore, assessments of genetic structure at a continental scale have also been carried out [67]. These studies have not only contributed to our understanding of the evolutionary history of European red deer but have also provided valuable insights into the impact of anthropogenic and environmental factors on population dynamics. Additionally, they contributed to conservation management framework of the European red deer. All these studies examining genetic structure and HFCs have been conducted using microsatellite markers. The advancements in SNP technology may offer new possibilities for the management of red deer populations. However, no study so far has compared the performance of both types of markers, microsatellite and SNP, for wild red deer populations. 

In this study, we aimed to compare the genetic structure and multilocus heterozygosity of wild red deer populations from Spain using 11 microsatellites and over 30,000 SNPs. Our objective was to determine the extent to which SNPs can offer better insights than microsatellites into the overall genetic status and population genetic processes. The comparisons encompassed genetic analyses commonly employed in wildlife management.

## 2. Materials and Methods

### 2.1. Study Area and Sample Collection

The study was conducted in six red deer populations located in various regions of Spain, including western, south-central, and northern areas (Figure S1). For western Spain, we included three populations, two of which were from the Sierra de San Pedro (SP1 and SP2; see [46]) and one from the Monfragüe National Park (MO). For south-central Spain, our study included two red deer populations from the Sierra Morena (SM1 and SM2; see [46,48]). These five red deer populations are situated within the native range of the Iberian red deer (*Cervus elaphus hispanicus*). For northern Spain, we included a population from the southern Pyrenees (PYS, see [38]). In PYS, red deer were introduced from various areas of the Iberian Peninsula, primarily from central Spain areas with populations genetically related to the SM2 [38]. 

We collected a piece of ear cartilage from 210 red deer culled by hunters. Sample sizes for each population were as follows: SP1 (*n* = 59), SP2 (*n* = 7), MO (*n* = 19), SM1 (*n* = 75), SM2 (*n* = 24), and PYS (*n* = 26). Specimens were hunted between 2020 and 2021 during regular sport hunting and management culling under local regulated hunting plans. This study never provoked hunters to shoot additional deer. 

### 2.2. DNA Extraction, Microsatellite and SNP Genotyping

We isolated genomic DNA from ear tags using the BioSprint^®^ 96 DNA Tissue Kit (Qiagen, Carlsbad, CA, USA) according to the manufacturer’s protocol. We genotyped the individuals with 12 microsatellite markers (BM1818, CP29, CSSM19, CSSM43, ETH225, FCB193, FCB304, FCB5, JP38, MM12, RME25, and TGLA53) according to the methodology described in [47]. We used positive and negative controls during the polymerase chain reactions. In all polymerase chain reactions, the positive control had the same genotype, and the negative control did not have amplification. Linkage disequilibrium (LD) for each pair of loci and departures from Hardy–Weinberg equilibrium (HWE) were assessed with Genepop 3.4 [68] (Table S1). No loci pair showed significant LD. Microchecker 2.2.3 [69] was used to determine the existence of null alleles, stuttering and large allele dropout. The CSSM43 marker was removed due to the existence of null alleles and a high proportion of missing data. The TGLA53 locus also had a relatively high proportion of missing data (Table S1), but repeating all the subsequent analyses with and without this marker did not alter the result. We present the results including TGLA53. 

For SNPs, the individuals were genotyped using the cervine 50K Illumina Infinium iSelect HD Custom BeadChip [70,71] including 50,841 SNPs. Plink [72] was used to detect and remove SNPs with more than 10% of missing data, linkage disequilibrium (variance inflation factor of 1.25), and less than 1% of minor allele frequencies, remaining 31.712 markers. The total genotyping rate was 0.993. The chromosome location and SNP position were referenced according to the assembly and annotation of the red deergenome version CerEla1.1 [73].

### 2.3. Genetic Analyses

The genetic diversity of each population was quantified with observed and expected heterozygosities (H_O_ and H_E_, respectively). Moreover, the relationship between both heterozygosities was determined with the F_IS_ parameter (inbreeding coefficient at population level). For microsatellites, genetic diversity measures were obtained with Genetix 4.05 [74]. For SNPs, these estimates were conducted with the dartR package [75] in R [76]. We calculated pairwise F_ST_ values and assessed their significance using Genetix (with 1000 permutations) for microsatellites and dartR (with 1000 bootstraps) for SNPs. Pairwise F_ST_ values obtained with both types of markers were compared with correlations and a Mantel test (10,000 permutations) in the vegan package [77]. Principal Component Analyses (PCAs) for both microsatellites and SNPs were conducted with the adegenet package [78]. The most probable number of genetic clusters in the study area, as well as the probability of assigning individuals to each cluster, were inferred with Structure 2.3.4 [79] for microsatellites. To determine the number of genetic clusters (K), ten independent runs of K = 1–7 were carried out with 500,000 iterations, following a burn-in period of 100,000 iterations. The Structure analyses utilized models of admixture ancestry and correlated allele frequencies. We used both the de novo and LOCPRIOR tests, and the results were similar. We present the results of the de novo test. We analyzed the model output using Structure Harvester [80] according to the ΔK method described in [81]. For SNPs, the most probable number of clusters was obtained by using the cross entropy method implemented in the LEA package [82]. LEA was also used to calculate the admixture coefficients of individuals.

We inferred the expected relationship between multilocus heterozygosity and inbreeding for both types of genetic markers using the inbreedR package [83]. InbreedR was used to estimate the identity disequilibrium parameter (g^2^), heterozygosity-heterozygosity correlation [26], and the expected r^2^ between multilocus heterozygosity and inbreeding. Standard deviations were obtained after 1000 permutations and 1000 bootstraps for g^2^, 1000 repetitions for HHC, and 1000 bootstraps for the expected r^2^. Finally, we quantified the correlation between multilocus heterozygosity obtained with both microsatellite and SNP markers.

## 3. Results

### 3.1. Genetic Diversity and Population Structure

**Figure 1:**
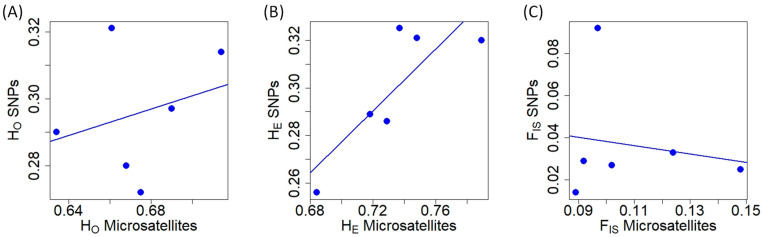
Relationship of different genetic diversity measures obtained with microsatellite and SNP markers in red deer populations. (**A**) Observed heterozygosity (H_O_). (**B**) Expected heterozygosity (H_E_). (**C**) Inbreeding coefficient at population level (F_IS_).

Regarding microsatellite genetic diversity at the population level, MO had the lowest H_O_, while SP2 showed the lowest H_E_ (Table 1). SM1 exhibited the highest heterozygosity (H_O_ and H_E_) at microsatellite markers. In terms of SNPs, SP2 displayed the lowest heterozygosity (H_O_ and H_E_), while the highest heterozygosity (H_O_ and H_E_) was observed in SM2 (Table 1). The populations with the lowest and highest F_IS_ values using microsatellites were SP2 and MO, respectively (Table 1). In contrast, with respect to SNPs, SP2 had the lowest F_IS_ value and PYS exhibited the highest one (Table 1). H_O_ obtained with microsatellites and SNPs did not exhibit a correlation (Pearson’s r = 0.280, t = 0.583, df = 4, *p* = 0.591; Figure 1A). The values of H_E_ from microsatellite and SNP markers were positively correlated (Figure 1B) (Pearson’s r = 0.828, t = 2.957, df = 4, *p* = 0.042). In terms of F_IS_, the values obtained with microsatellites and SNPs were not significantly related (Pearson’s r = −0.164, t = −0.332, df = 4, *p* = 0.757; Figure 1C). H_O_ and H_E_ were not correlated for microsatellites (Pearson’s r = 0.641, t = 1.669, df = 4, *p* = 0.170; Figure S2A), but they were significantly correlated for SNPs (Pearson’s r = 0.904, t = 2.242, df = 4, *p* = 0.013; Figure S2B). In the case of microsatellites, the lack of correlation was mainly due to one population (SP2; Figure S2A). 

**Figure 2:**
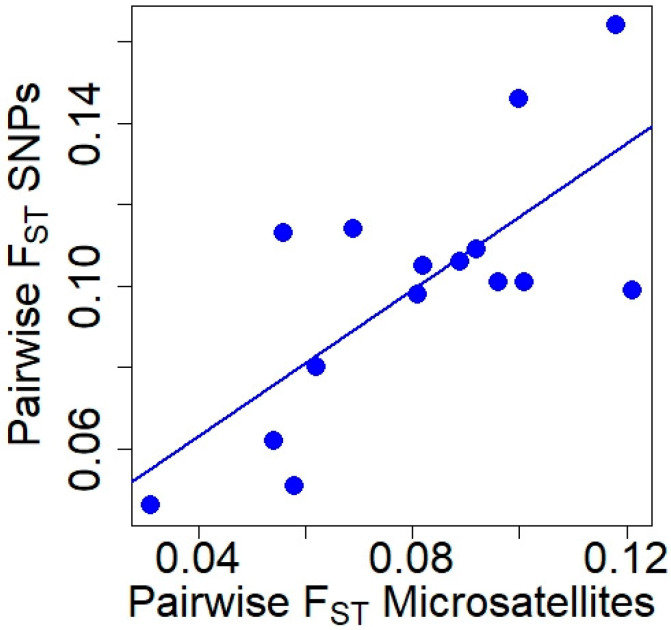
Relationship between pairwise F_ST_ values obtained with microsatellite and SNP markers in red deer populations.

Pairwise F_ST_ values obtained with microsatellite and SNP markers (Table 2) were positively correlated (Pearson’s r = 0.726, t = 3.805, df = 13, *p* = 0.002; Figure 2), although the Mantel test did not reach significance (Mantel statistical r = 0.519, *p* = 0.067). The genetic differentiation between all pairs of populations was significant for both types of markers (*p* ≤ 0.001). 

**Figure 3:**
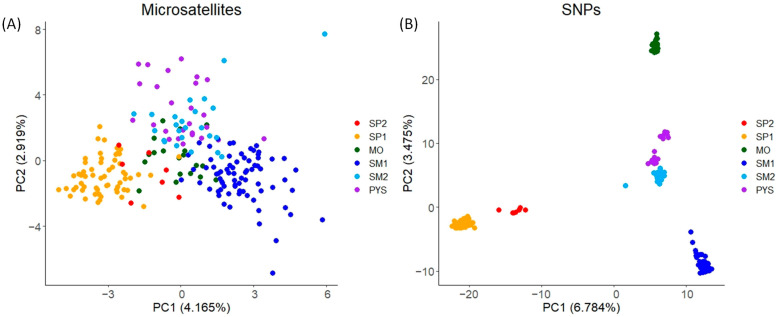
PCA plots for the two PCs with the highest eigenvalues obtained in red deer populations. (**A**) PCA obtained with microsatellite markers. (**B**) PCA obtained with SNPs. The figure shows the percentage of the total variance explained by each PC (in brackets). SP2: Sierra de San Pedro 2. SP1: Sierra de San Pedro 1. MO: Monfragüe National Park. SM1: Sierra Morena 1. SM2: Sierra Morena 2. PYS: southern Pyrenees. Order of populations: west to east (see Figure S1).

In the PCA obtained with microsatellites, individuals from the same populations tended to have similar PC1 and PC2 scores (mainly individuals from SP1 and SM1; Figure 3A). These scores displayed relatively low variation among populations (Figure 3A). Conversely, for SNPs, the PCA scores exhibited higher variation, with PC1 mainly differentiating SP1 and SM1, and PC2 mainly differentiating MO from the remaining populations (Figure 3B).

**Figure 4:**
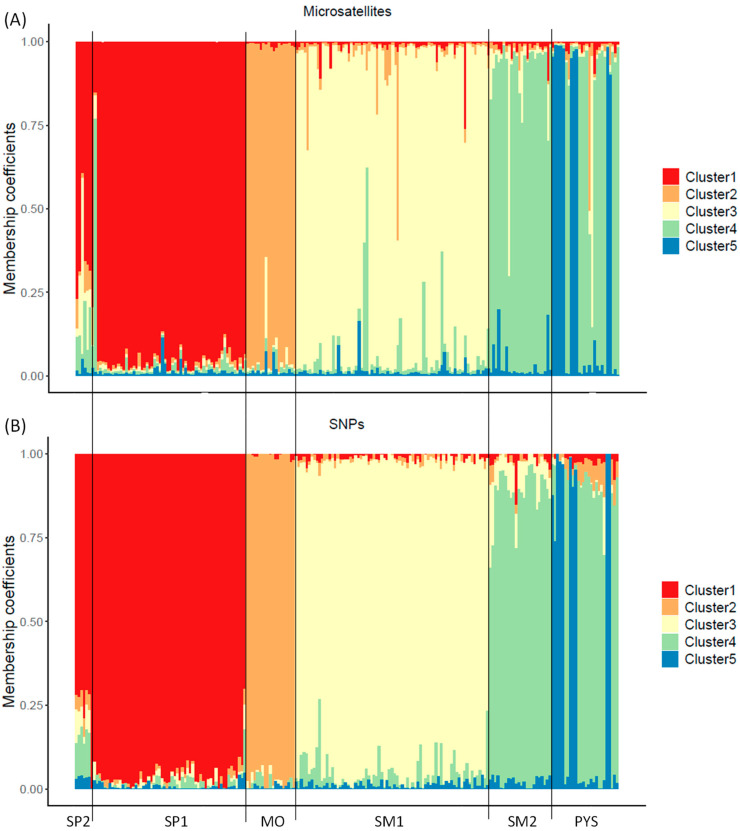
Membership/ancestry coefficients obtained with microsatellite (**A**) and SNP markers (**B**) for K = 5 in red deer populations. Each individual is represented by a thin vertical line, which is portioned into 5 segments with different colors representing the individual’s estimated membership fraction in K clusters. SP2: Sierra de San Pedro 2. SP1: Sierra de San Pedro 1. MO: Monfragüe National Park. SM1: Sierra Morena 1. SM2: Sierra Morena 2. PYS: southern Pyrenees. Order of populations: west to east (see Figure S1).

From the Structure analyses, ΔK showed that K = 2 was the most probable number of clusters in the studied populations (highest ΔK; Figure S3A). However, for the SNP assay, the cross-entropy results showed that K = 5 was the most probable number of genetic clusters (lowest cross-entropy; Figure S3B). Despite the Structure analyses with microsatellites not resulting in K = 5 as the most probable number of clusters, the membership coefficients (q_i_) were similar to the ancestry coefficients obtained with SNPs (Figure 4 and Figure S4). Similar results with both genetic markers were also obtained for K = 2 (Figures S5 and S6). However, intrapopulation membership/ancestry coefficients had higher variation for microsatellites than for SNP markers: individuals with similar ancestry coefficients (SNP analysis) had different membership coefficients (microsatellite analysis), mainly at intermediate values (Figures S4 and S6). 

### 3.2. Multilocus Heterozygosity and Inbreeding

**Figure 5:**
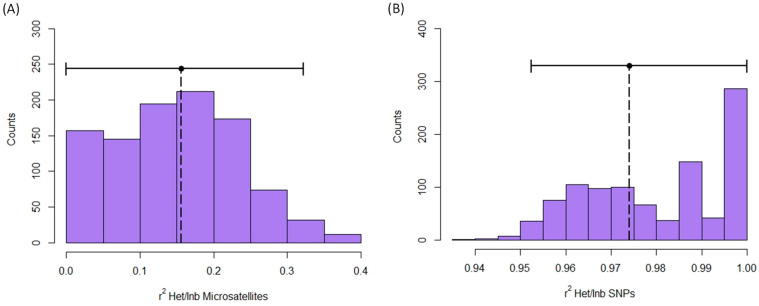
Expected r^2^ between inbreeding and multilocus heterozygosity obtained with microsatellites (**A**) and SNPs (**B**) in red deer populations. Figure shows the histogram of r^2^ values in permutations. Observed r^2^ is represented by a vertical dotted line. Horizontal continuous lines represent the confidence intervals.

Identity disequilibrium was not significantly different from 0 when it was quantified with microsatellites (g^2^ = 0.008, S.E. = 0.005, C.I. (5%/95%): −0.002/0.018; Figure S7A). Contrarily, identity disequilibrium with SNPs was significantly greater than 0 (g^2^ = 0.0049, S.E. = 0.0005, C.I. (2.5%/97.5%): 0.0038/0.0059; Figure S7B). Moreover, the heterozygosity-heterozygosity correlation was only significantly different from 0 for SNPs (microsatellites: HHC = 0.096, S.D. = 0.049, C.I. (5%/95%) = −0.006/0.187, Figure S8A; SNPs: HHC = 0.974, S.D. = 0.003, C.I. = 0.969/0.979, Figure S8B). Finally, the expected r^2^ between inbreeding level and multilocus heterozygosity (MLH) was only significantly greater than 0 for SNPs (Microsatellites: r^2^ = 0.164, C.I. = 0.000/0.314, Figure 5A; SNPs: r^2^ = 0.974, C.I. (5%/95%) = 0.951/1.000, Figure 5B).

**Figure 6:**
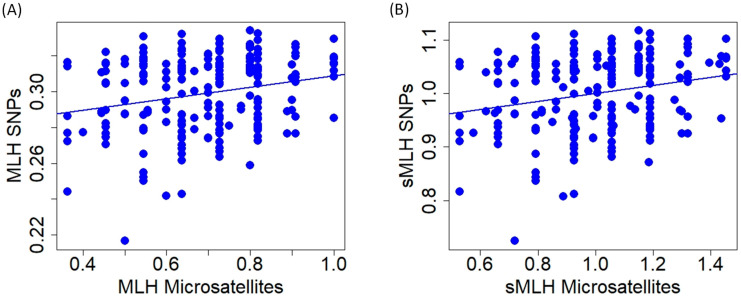
Relationship of multilocus heterozygosity (**A**); (MLH) and standardized multilocus heterozygosity (**B**); (sMLH) of red deer individuals obtained with microsatellite and SNP markers.

At the individual level, multilocus heterozygosity and standardized multilocus heterozygosity obtained with microsatellites and SNPs were significantly correlated, but relationships show high variability (MLH: r = 0.234, t = 3.480, *p* < 0.001; sMLH: r = 0.228, t = 3.382, *p* < 0.001; Figure 6).

## 4. Discussion

In the case of red deer, we found that the SNPs improved the assessment of specific parameters in relation to microsatellites, particularly in those analyses related to the distribution of genetic diversity across individuals. Nonetheless, our results revealed statistically significant correlations between certain parameters quantified using both types of genetic markers.

In our red deer populations, two parameters related to the genetic structure were correlated when we used microsatellites and SNPs: H_E_ and pairwise F_ST_ values. Therefore, microsatellite markers captured the amount of genetic diversity within the studied populations as well as the genetic differentiation among populations. Contrarily, the comparison in H_O_ and F_IS_ quantified with microsatellites and SNPs exhibited low correlation. Accordingly, the limited number of microsatellites might have low precision in detecting how the genetic diversity in the population is distributed across the individuals in our studied red deer populations. Similar correlations between the assessed parameters with both types of markers have been observed in other species, such as the Gunnison sage-grouse (*Centrocercus minimus*, [33]) and the Atlantic salmon (*Salmon salar*; [84]). However, studies with similar objectives in other species did not show the same correlations between the estimated parameters (e.g., [23] with *Arabidopsis halleri*, [85] with *Armillaria cepistipes*). Therefore, our results indicate that the relationship between the parameters describing the genetic structure of populations obtained with microsatellites and SNPs varies depending on the studied species. Moreover, we cannot rule out the potential impact of sample sizes (number of populations and number of individuals per population), which might differentially affect the correlation of each genetic parameter [24]. In this sense, the small sample size in SP2 might impact the observed correlations. For instance, since H_E_ is sample size dependent, the small sample size might contribute to the low H_E_ in SP2. Therefore, it might affect the low correlations between H_O_ and H_E_ at microsatellites. However, the absence of correlations between genetic measurements obtained with microsatellites and SNPs was not limited to one population (Figure 1A,C). 

We found differences in the principal component analysis obtained with both types of markers, indicating high levels of genetic differentiation for SNPs compared to the results with microsatellites. Despite these differences, the percentages of explained variance of PC1 and PC2 were low in both types of markers. The low percentages might be due to the lower capacity to detect population-specific genetic variations of microsatellites and the noise provided by irrelevant SNPs. The genetic differentiation observed in the PCA, obtained with SNPs, was further supported by the admixture analysis conducted using SNPs. The cross-entropy analysis in the admixture procedure identified five clusters as the most likely number of genetic groups. The number of clusters and the membership scores can be readily discerned in the PCA obtained using SNPs. In contrast, with the microsatellites, the genetic structure is less discernible in the PCA plot, and the ΔK procedure applied to the Structure results suggested the existence of a smaller number of clusters. Similar discrepancies in the PCA plots between markers were observed in the Mediterranean tortoise (*Testudo hermanni*, [86]). Variations in the optimal number of clusters were also detected in amphibians (*Hyla molleri* and *Pelobates cultripes*, [87]). Therefore, our findings in red deer support that SNPs can provide additional insights into the genetic substructure of populations. On the other hand, the distribution of membership coefficients across individuals was similar for both microsatellites and SNPs, although the within-population variation of these values was higher when microsatellites were used. This result suggests the existence of risks when using microsatellites for assigning admixture or introduction/translocations of specific individuals in a population (see, e.g., [49,88]). Therefore, there is a clear need to develop approaches that leverage SNP technology to enhance the accuracy and reliability of such analyses.

Concerning the utility of using multilocus heterozygosity at the individual level, we obtained positive values for g^2^, HFC and expected r^2^ with inbreeding when microsatellites were used. However, the C.I. of these values included zero. Contrarily, the positive values of these parameters at SNPs did not include zero, even HHC and expected r^2^ values were close to one. Despite the significant relationship between multilocus heterozygosity in our sample of red deer at both genetic markers, the use of a high number of SNPs represents a clear advantage in the study of inbreeding in natural populations of red deer. Similarly, Miller et al. [89] reported high differences in the estimates of g^2^ and r^2^ between small numbers of microsatellites and a large number of SNPs. Thus, despite the existence of studies that have detected HFCs with microsatellites in red deer [44,45], the utilization of SNPs holds great potential in the investigation of processes related to inbreeding in natural populations. These advantages in detecting HFC and inbreeding-related processes are expected under all the hypotheses that explain the relationship between heterozygosity and fitness [90].

## 5. Conclusions

The results regarding the genetic structure of populations and the multilocus heterozygosity of the individuals, obtained with microsatellite markers, generally correlated with those obtained using SNPs. Despite the heterogeneous sample sizes and the limited number of red deer populations, microsatellites provided insights into the overall genetic status and the existence of population processes in red deer. Nevertheless, the greater precision in inferring genetic structure and the increased power to detect HFCs encourage scientists and wildlife managers to use SNPs wherever possible. In our red deer populations, the utilization of SNPs revealed patterns that were not apparent in the analyses conducted with microsatellites. Moreover, SNP technology holds high potential for detecting inbreeding events in a species like red deer, which has important ecological, health, and economic implications.

## Figures and Tables

**Table 1:** Genetic diversity of red deer populations for microsatellite and SNP markers. SP2: Sierra de San Pedro 2. SP1: Sierra de San Pedro 1. MO: Monfragüe National Park. SM1: Sierra Morena 1. SM2: Sierra Morena 2. PYS: southern Pyrenees. Order of populations: west to east (see Figure S1). All: sum of sample sizes in N; mean values in the remaining variables.

		Microsatellites			SNPs		
Pop	N	H_O_	H_E_	F_IS_	H_O_	H_E_	F_IS_
SP2	7	0.675	0.684	0.089	0.272	0.256	0.014
SP1	59	0.668	0.729	0.092	0.280	0.286	0.029
MO	19	0.634	0.718	0.148	0.290	0.289	0.025
SM1	75	0.714	0.789	0.102	0.314	0.320	0.027
SM2	24	0.661	0.737	0.124	0.321	0.325	0.033
PYS	26	0.690	0.748	0.097	0.297	0.321	0.092
All	210	0.674	0.734	0.109	0.296	0.299	0.037

**Table 2:** Pairwise F_ST_ values calculated using microsatellites (above the diagonal) and SNPs (below the diagonal) in the studied red deer populations. SP2: Sierra de San Pedro 2. SP1: Sierra de San Pedro 1. MO: Monfragüe National Park. SM1: Sierra Morena 1. SM2: Sierra Morena 2. PYS: southern Pyrenees. Order of populations: west to east (see Figure S1).

	SP2	SP1	MO	SM1	SM2	PYS
SP2		0.062	0.118	0.069	0.092	0.056
SP1	0.080		0.100	0.096	0.081	0.082
MO	0.164	0.146		0.089	0.121	0.101
SM1	0.114	0.101	0.106		0.058	0.054
SM2	0.109	0.098	0.099	0.051		0.031
PYS	0.113	0.105	0.101	0.062	0.046	

## Data Availability

The data presented in this study are available on request from the corresponding author.

## Supplementary Materials

The following supporting information can be downloaded at: https://www.mdpi.com/article/10.3390/ani13213374/s1, Figure S1: Locations of red deer populations in Spain; Table S1: Description of 11 microsatellite markers used for genotyping red deer in this study; Figure S2: Relationship between H_O_ and H_E_ for microsatellites and SNPs in red deer populations. Figure S3: Probability of the assessed number of genetic clusters (K) in red deer; Figure S4: Relationship between membership/ancestry coefficients obtained with both microsatellites and SNPs for each of the five clusters in red deer populations; Figure S5: Membership/ancestry coefficients obtained with both microsatellite and SNP markers for K = 2 in six red deer populations; Figure S6: Relationship between membership/ancestry coefficients obtained with both microsatellites and SNPs for the first cluster at K = 2 in six red deer populations; Figure S7: Identity disequilibrium (g^2^) obtained with microsatellite and SNP markers in red deer populations; Figure S8: Heterozygosity-heterozygosity correlation (HHC) obtained with microsatellites and SNPs in red deer populations.
