# Supplementary material for: Comparative Analysis of Microsatellite and SNP Markers for Genetic Management of Red Deer
Source: Animals (Basel). 2023 Oct 31;13(21):3374. doi: 10.3390/ani13213374 (PMC10650148; doi:10.3390/ani13213374)
Supplement: Supplementary file 1 [file animals-13-03374-s001.zip › animals-2654302-supplementary.pdf]

Supplementary Material

Comparative Analysis of Microsatellite and SNP Markers for Genetic Management of Red Deer

Javier Pérez-González [1,*], Juan Carranza [2], Gabriel Anaya [2,3], Camilla Broggini [2], Giovanni Vedel [2], Eva de la Peña [2,4] and Alberto Membrillo [2,5]

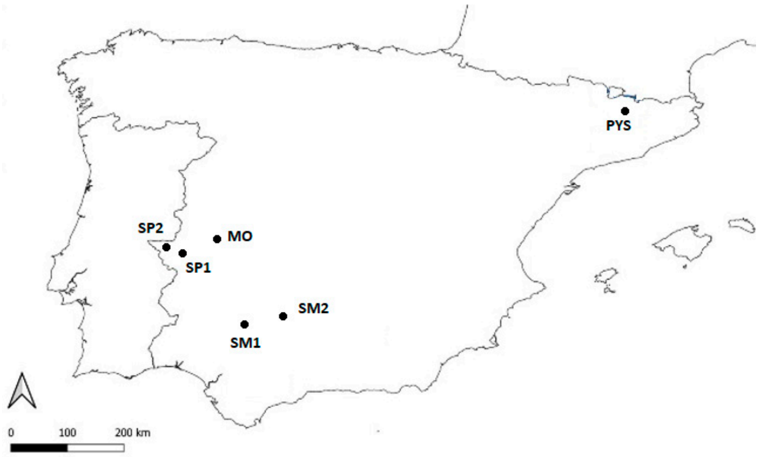

Figure S1. Locations of red deer populations in Spain. SP2: Sierra de San Pedro 2. SP1: Sierra de San Pedro 1. MO: Monfragüe National Park. SM1: Sierra Morena 1. SM2: Sierra Morena 2. PYS: southern Pyrenees. Order of populations: west to east.

Table S1. Description of 11 microsatellite makers used for genotyping red deer in this study. HWE: number of populations where the locus was in Hardy-Weinberg equilibrium. A: number of alleles. Range: range of allele sizes (base pairs). Missing: percentage of missing data.

Loci	HWE	A	Range	Missing
BM1818	4	10	232-252	0
CP26	6	19	130-176	2.38
CSSM19	4	12	129-155	0
ETH225	6	16	138-170	0
FCB193	3	21	100-150	6.67
FCB304	6	12	118-146	0.48
FCB5	6	14	184-212	4.29
JP38	6	15	201-243	0
MM18	6	6	181-197	0.95
RME25	6	9	147-207	0
TGLA53	3	17	150-202	26.19

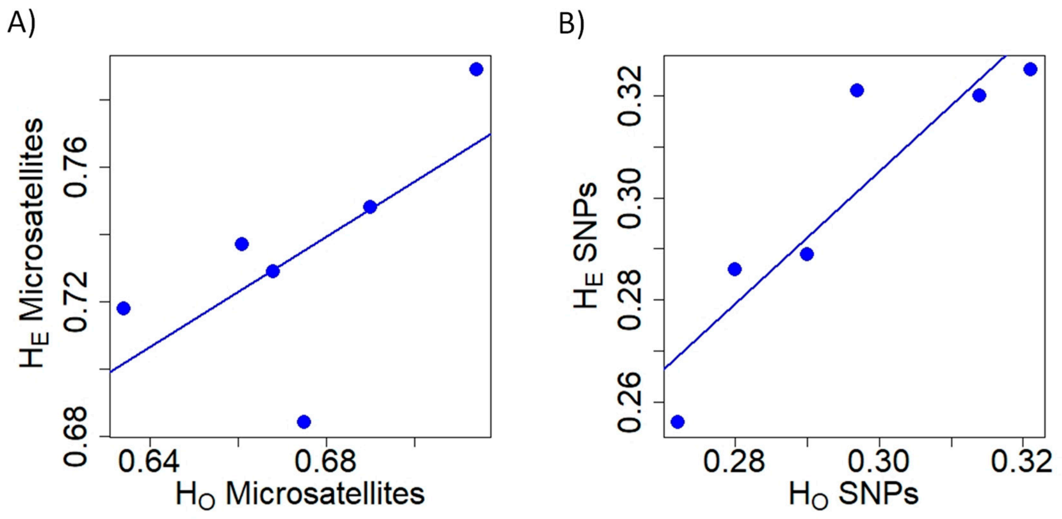

Figure S2. Relationship between observed heterozygosity (H_O) and expected heterozygosity (H_E) for microsatellites (A) and SNPs (B) in red deer populations.

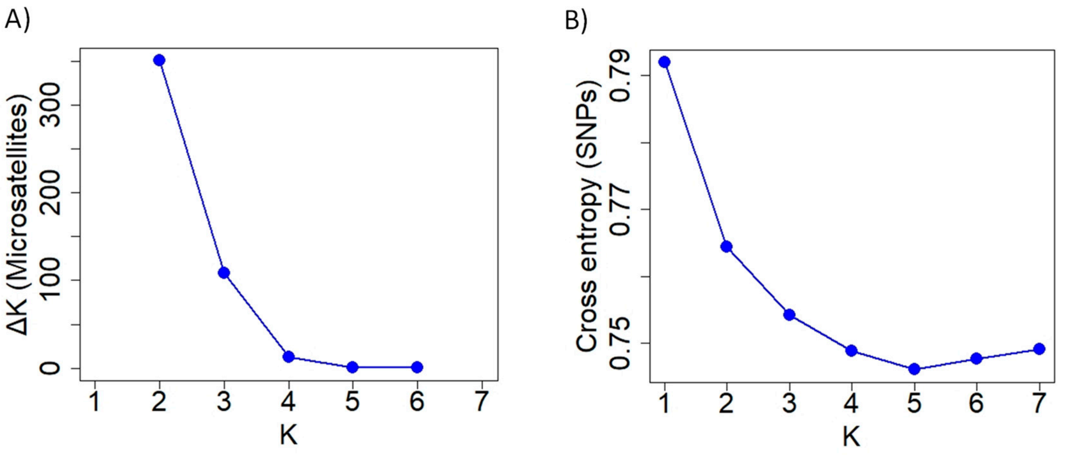

Figure S3. Probability of the assessed number of genetic clusters (K) in red deer. The figure shows the ΔK values for microsatellite data (A) and cross-entropy values for SNP data (B).

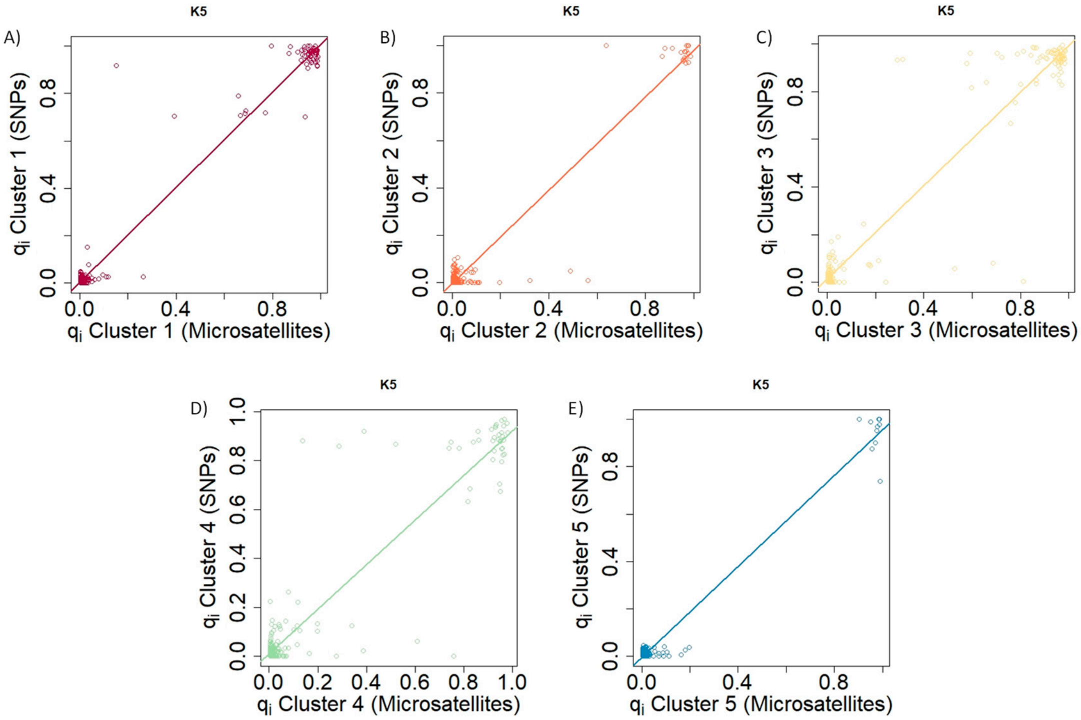

Figure S4. Relationship between membership/ancestry coefficients (q_i) obtained with both microsatellites and SNPs for each of the 5 clusters (cluster 1 (A), cluster 2 (B), cluster 3 (C), cluster 4 (D), and cluster 5 (E)) at K = 5 in red deer populations.

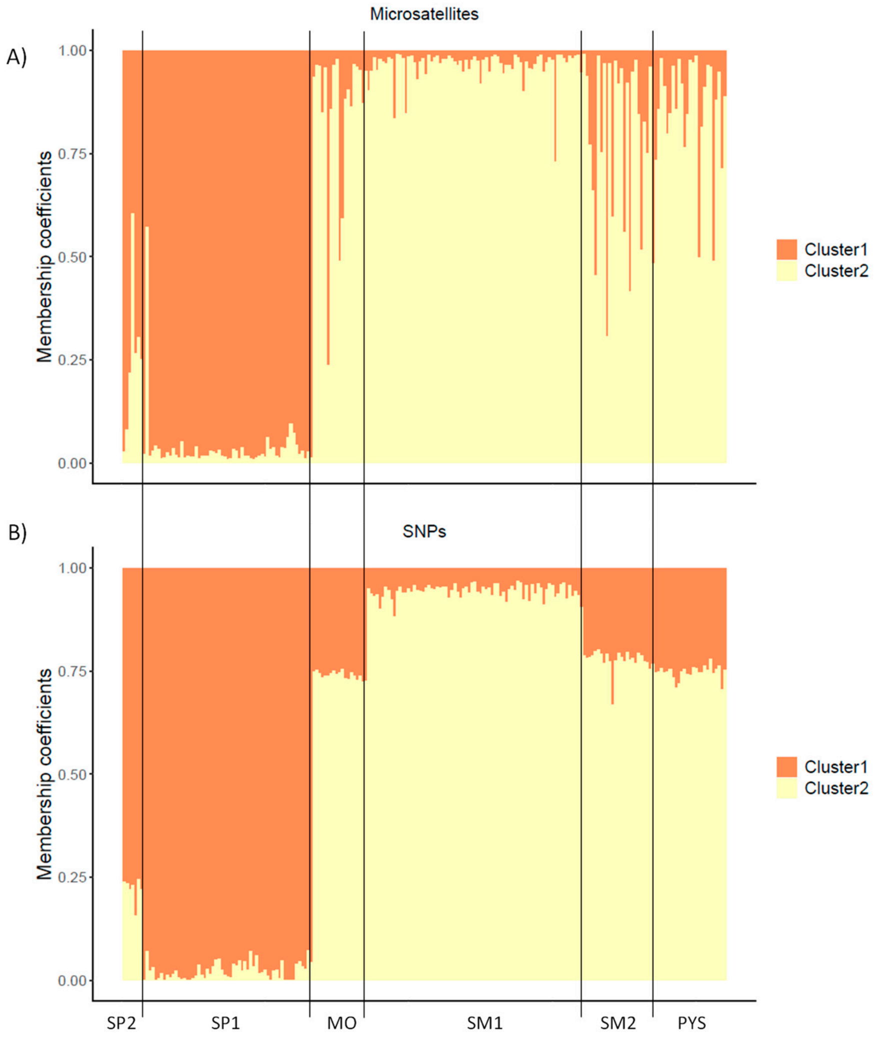

Figure S5. Membership/ancestry coefficients obtained with microsatellite (A) and SNP (B) markers for K = 2 in six red deer populations. Each individual is represented by a thin vertical line, which is portioned into 2 segments with different colors representing the individuals' estimated membership fraction in K clusters. SP2: Sierra de San Pedro 2. SP1: Sierra de San Pedro 1. MO: Monfragüe National Park. SM1: Sierra Morena 1. SM2: Sierra Morena 2. PYS: southern Pyrenees. Order of populations: west to east (see Figure S1).

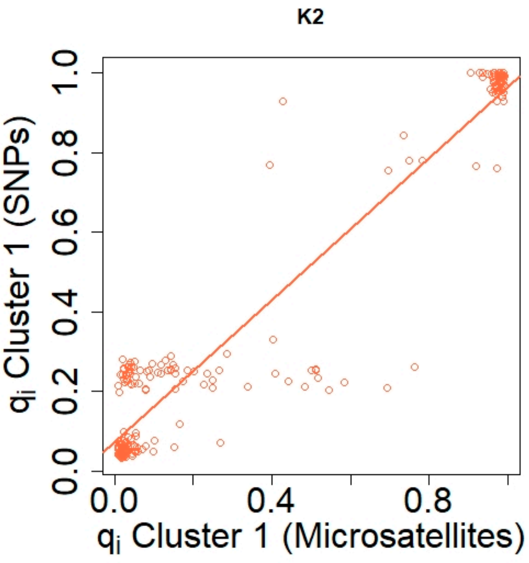

Figure S6. Relationship between membership/ancestry coefficients (q_i) obtained with both microsatellites and SNPs for the first cluster at K = 2 in six red deer populations.

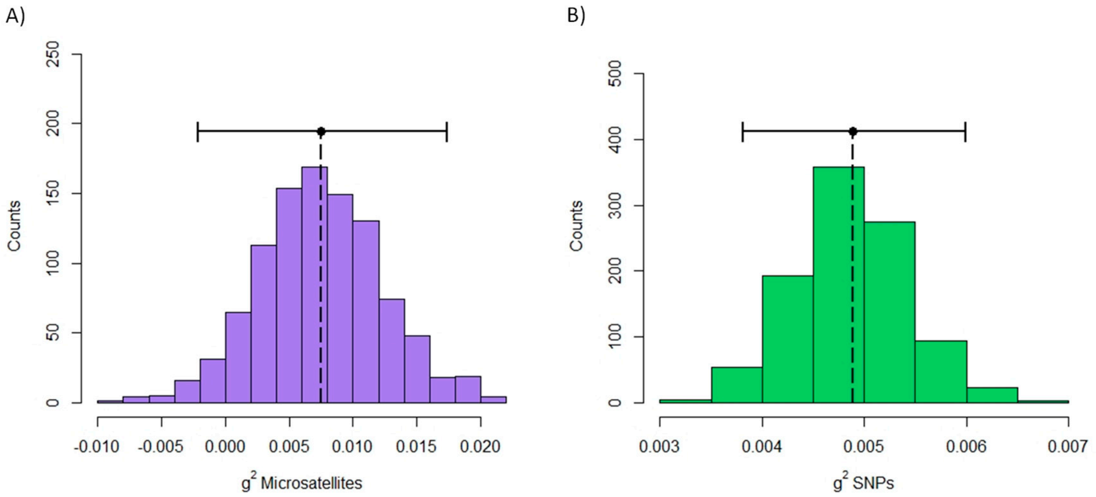

Figure S7. Identity disequilibrium (g^2) obtained with microsatellite (A) and SNP (B) markers in red deer populations. Observed g^2 is represented by a vertical dotted line. Confidence interval is represented by a horizontal continuous line.

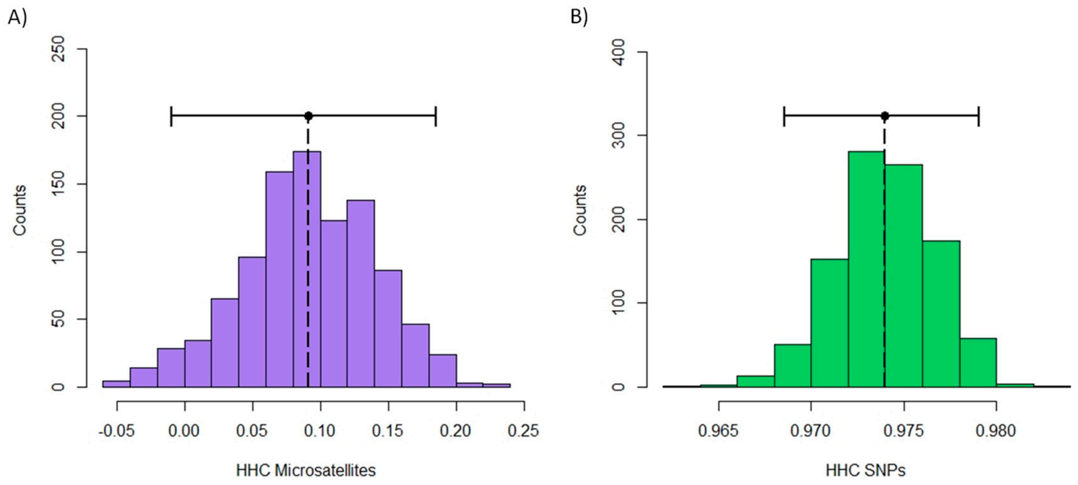

Figure S8. Heterozygosity-heterozygosity correlation (HHC) obtained with microsatellites (A) and SNPs (B) in red deer populations. Figure shows the histogram of HHC values in permutations. Observed HHC is represented by a vertical dotted line. Confidence interval is represented by a horizontal continuous line.
